# The relationship between social support, e-Health literacy, sleep quality and Internet addiction disorder in college students: a cross-sectional study

**DOI:** 10.3389/fpsyg.2026.1754039

**Published:** 2026-04-07

**Authors:** Wenwen Zhang, Xingmei Yin, Weijing Zhang, Yanan Wang, Xiuyun Wang

**Affiliations:** 1 Dongying People’s Hospital (Dongying Hospital of Shandong Provincial Hospital Group), Dongying, China; 2 Dingzhuang Central Health Center, Dongying, China

**Keywords:** college students, e-Health literacy, Internet addiction disorder, sleep quality, social support

## Abstract

**Background:**

The widespread use of the Internet has increased the risk of Internet addiction disorder (IAD), particularly among university students. This study aims to investigate the relationship between social support and Internet addiction disorder, with a focus on the mediating roles of e-Health literacy and sleep quality.

**Methods:**

A cross-sectional survey was conducted among university students in Nantong, China, using simple random sampling. Valid data were collected from 774 participants (response rate: 95.51%). Measures included sociodemographic characteristics, Internet Addiction Test (IAT), Perceived Social Support Scale (PSSS), e-Health Literacy Scale (e-HEALS), and Pittsburgh Sleep Quality Index (PSQI). Structural equation modeling (SEM) with bootstrap testing was employed to examine the multiple mediation effects.

**Results:**

The baseline score for Internet addiction disorder was 38.16 (SD = 0.48). Social support, e-Health literacy, poor sleep quality, and Internet addiction disorder were significantly negatively related to each other. All paths in the research model were statistically significant, and social support cognition level was an important factor affecting Internet addiction disorder, and the direct effect accounted for 45.60% of the total effect. The total indirect effect of social support through e-Health literacy and sleep quality on Internet addiction disorder was statistically significant.

**Conclusion:**

Enhancing social support, improving e-Health literacy, and promoting better sleep quality may effectively reduce Internet addiction disorder among university students. These findings provide a theoretical basis for targeted interventions.

## 1 Introduction

With the rapid development of information technology, the use of the Internet has become more widespread, which can provide various entertainment and social networking opportunities (Chou et al., 2005; Chou et al., 2015) to meet the needs of individual life. However, more and more evidence shows that the way and use of the Internet will determine the advantages and disadvantages of the Internet (Chu et al., 2017). The importance of using the Internet reasonably and correctly has also received increasing attention, and the blind and excessive use of the Internet will also become a problem that cannot be ignored under the development of information technology. Relevant studies have confirmed the emergence of Internet addiction caused by improper use of the Internet (Baturay and Toker, 2019). Internet addiction disorder refers to pathological Internet use as uncontrollable and problematic use of the Internet (Akhter, 2013), which is a growing issue in clinical and public health perspectives (Christakis and Moreno, 2009). Meanwhile, the phenomenon of Internet addiction can bring a series of harms to an individual’s life, study, and work (Gao et al., 2025). How to reduce an individual’s Internet addiction disorder has become an inevitable topic.

A large amount of research has demonstrated that youths are more susceptible to Internet addiction disorder compared with others in society (Jang et al., 2008); furthermore, the Internet has been an important part of student life (Chou et al., 2005). Meanwhile, college students had more time and more advanced electronic equipment to use the Internet than other people (Chi et al., 2016), and they had a higher ability to learn to use the Internet. However, college students demonstrated poor ability to discriminate and self-control (Briones, 2015; Sawyer et al., 2009), making them susceptible to temptation when encountering new and exciting Internet information and failing to consider the physical and mental harm it may cause (Davis, 2001; Ha et al., 2007). A study suggests that Chinese college students may be susceptible to problematic Internet use (Kandell, 1998). Further, a comprehensive meta-analysis in China showed that the prevalence of Internet addiction disorder was from 1.9 to 49.41% among Chinese university students (Li et al., 2018; Shao et al., 2018). And many studies have reported an increasing prevalence of Internet addiction disorder (Kuss et al., 2014). So, it is necessary to study the Internet addiction disorder of college students in China.

### 1.1 The relationship between social support and internet addiction disorder

Previous studies revealed that social factors for the formation of Internet addiction disorder played a certain role, such as social skills (Ghassemzadeh et al., 2008), social isolation (Kuss et al., 2014), social function (De Leo et al., 2013), interpersonal relationships (Li and Chung, 2006), and peer contagion (Pan and Yeh, 2018). The Self-Determination theory (Deci and Ryan, 1985) and the Need-to-Belong theory (Baumeister et al., 1995) emphasize that the survival and development of individuals require recognition and connection with friends, family, organizations, and society, and its recognition and connection are crucial for emotional and social adjustment. When students receive adequate social support from family, peers, and mentors, their need for relatedness is fulfilled in real-life contexts, reducing the motivation to seek compensatory belongingness through excessive online engagement. Meanwhile, when students have trusted others to turn to during emotional distress, they are less likely to use the Internet as a maladaptive coping strategy. Social support is the basis of individual social networks (Whittaker and Garbarino, 1983), which provide more ways for individuals to connect, which can give more companionship and support to college students (Fondacaro and Heller, 1983), as well as help them get rid of the dilemma of survival and development (Wu and Cheng, 2007). Some studies have contributed to the growing evidence of the relationship between social support and Internet addiction disorder in recent years. Studies have revealed that social support offline has a negative relationship with Internet addiction disorder (Wu et al., 2016). College students with good social support had strong social, personal, and familial relationships (Brannan et al., 2013), which could reduce the prevalence of Internet addiction disorder through assistance from their social networks (Mo et al., 2018). Concomitantly, close social bonds are invariably associated with rich offline social interactions, cultural engagements, and shared goals (Lieberman and Schroeder, 2020). These healthy alternative pursuits not only occupy individuals’ leisure time but also deliver authentic interactive experiences and a sense of accomplishment that the virtual world cannot fully replicate (Turner, 2022). Hence, we propose that social support is negatively associated with Internet addiction disorder.

### 1.2 The relationship between social support, e-Health literacy, and sleep quality

Undoubtedly, with the steady rise in global internet penetration rates, the internet has become a primary medium for college students’ daily communication and social interaction, as well as a key resource for their access to health-related information and knowledge (Wang et al., 2024a). And e-Health literacy (Norman and Skinner, 2006a), conceptualized as the dimension of health literacy specific to internet-based health information seeking and use, is defined as the ability to search for, comprehend, and apply health-related knowledge via the internet to advance individual health and wellbeing. According to Social Cognition Theory (Bandura, 2001), key individuals within social networks (e.g., peers and professional educators) can enhance students’ perceptions of the threats posed by internet addiction—specifically its perceived susceptibility and severity—by disseminating authoritative health-related knowledge, recommending credible online resources (websites and applications), and raising awareness of healthy internet use (Wang et al., 2024b). Concomitantly, these individuals can actively acquire health information, thereby motivating adaptive behavioral change toward regulated internet use. Meanwhile, some studies have shown that the main behavioral manifestations of Internet addiction disorder, are excessive use of the Internet (Yellowlees and Marks, 2007) (for example: spending a lot of time online every day, and the online time is constantly increasing, making it difficult to control the online duration), Moreover, Time Displacement Hypothesis (Robinson et al., 2002) posits that when an individual’s available time is fixed, the more time spent online, the less time spent on other activities. Increasing evidence has also confirmed that college students cannot control their online time (Röhlke, 2025). The mentality of “playing another round” or “watching another video” can lead to habitual staying up late, which seriously affects sleep quality. Based on the Health Action Process Approach (HAPA) (Schwarzer, 2016), eHealth literacy influences sleep quality through the “motivation-intention-action control” behavioral change pathway, thereby ultimately mitigating the risk of Internet addiction disorder. In the motivational phase, individuals with high eHealth literacy recognize that excessive internet use impairs sleep regularity and poses adverse effects on physical health, while concurrently enhancing their knowledge of sleep hygiene. In the action control phase, these individuals can develop specific implementation intentions (e.g., setting pre-sleep digital disconnection reminders) to achieve the goal of regulating excessive internet use. Meanwhile, Social Control Theory (Hirschi and Stark, 1969) posits that members within an individual’s social network exert normative influence, behavioral constraints, and supervisory oversight over their behaviors through expressions of care, timely reminders, and normative expectations. This multifaceted social regulation guides individuals to adhere to healthy daily (such as regular work and rest, reasonable online time, etc.) and mitigate inappropriate internet use. Based on the analysis, we propose that social support is positively associated with e-Health literacy and sleep quality, and social support exerts an indirect positive influence on sleep quality through e-Health literacy.

### 1.3 The relationship between social support, e-Health literacy, sleep quality, and Internet addiction disorder

**Figure 1:**
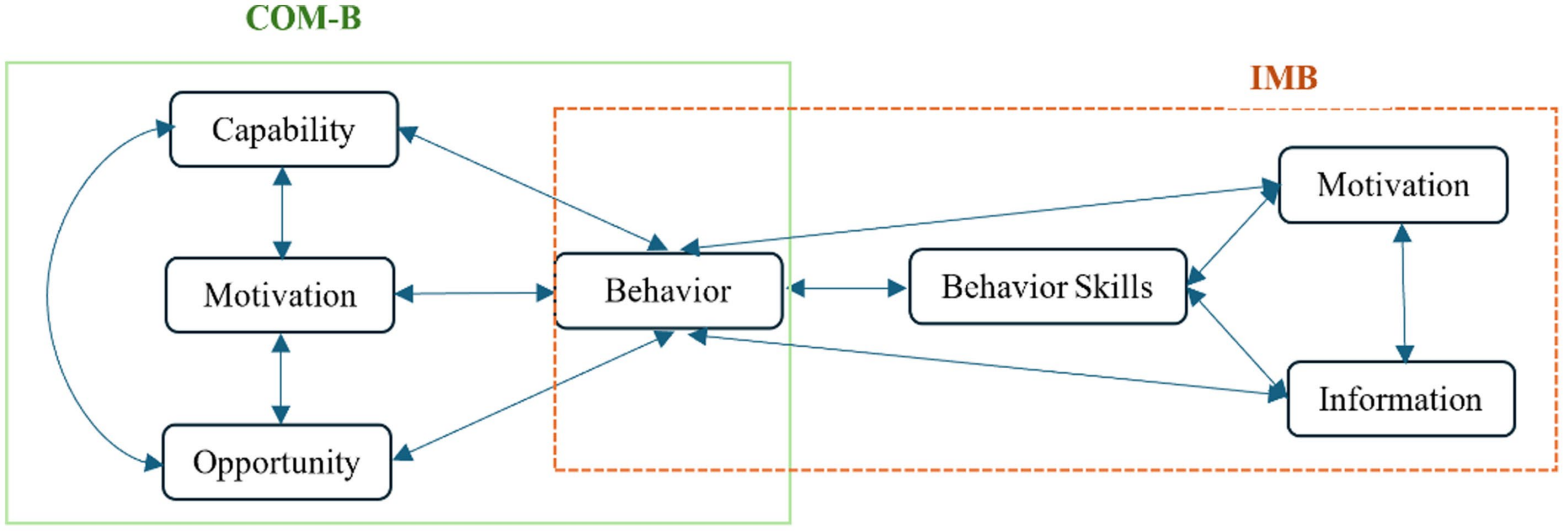
Capability, Opportunity, Motivation, and Behavior model (COM-B) and Information-Motivation-Behavioral Skills Model (IMB).

**Figure 2:**
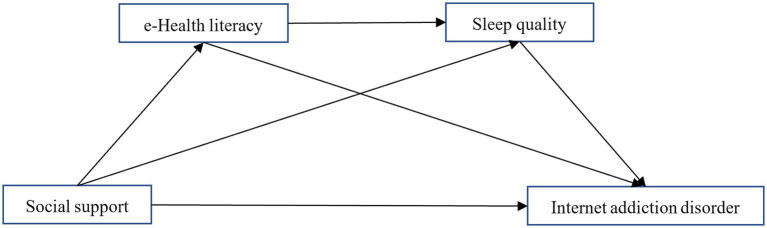
A multivariate mediating model between social support, e-Health literacy, sleep quality, and Internet addiction disorder.

Based on the Capability, Opportunity, Motivation, and Behavior model (Michie et al., 2011) (COM-B) and Information-Motivation-Behavioral Skills Model (IMB) (Fisher and Fisher, 1992) in Figure 1, guiding college students to use the Internet moderately hinges on systematically building the Information, Capability, Opportunity, and Motivation necessary for healthy online behavior. E-health literacy, as one of the core capabilities in online usage, enables individuals to effectively obtain, understand, and apply online health information (Norman and Skinner, 2006a), and thereby reasonably control their online time (Hanik, 2011). The cultivation of this capability is related social opportunities and information provided by social support: interpersonal relationship networks not only offer channels for individuals to obtain information (Eng et al., 1998), but also assist them in screening and identifying information in the vast amount of data (Cohen and Syme, 1985), and provide crucial support for the formation of healthy behaviors (Sallis et al., 1987; Wu et al., 2023). Ultimately, this positive behavioral transformation—driven by Information, Capability, Opportunity, and Motivation—facilitates the translation of individuals’ eHealth literacy into specific behavioral skills and health-promoting behaviors, such as sleep quality improvement (Kim and Oh, 2021). Thus, amid the unavoidable prevalence of internet use (Ghassemzadeh et al., 2008), individuals can achieve a transition from passive immersion to active self-regulation, thereby attaining the goal of moderate and beneficial internet engagement. Self-determination theory (Deci et al., 2017) also indicates that individuals receive the information and support of the outside world, out of respect for their interests, which would promote a change of behavior. In short, based on the existing research status, this study tries to construct a multivariate mediating model and path model with social support as the independent variable and electronic health literacy and sleep quality as the mediating variables to deeply explore the relationship between social support, e-Health literacy and sleep quality and college students’ Internet addiction disorder (Figure 2), providing a new perspective for understanding and intervening in college students’ Internet addiction disorder.

## 2 Methods

### 2.1 Study design

In this study, which combines multiple aspects such as economic development level, population distribution, and geographical accessibility, Jiangsu Province was selected as the investigation site, and a comprehensive university (encompassing various majors) was chosen using random selection. Additionally, a pre-survey was conducted before the official start of this study to refine the questionnaire and determine the prevalence rate of Internet addiction disorder. The minimum sample size required for the research was obtained through the software Raosoft online sample size calculator, with a confidence interval of 95% and an error margin of 3% included. Combined with the pre-survey results, the prevalence rate of Internet addiction disorder was 22.18%. The number of students currently enrolled in this university is nearly 50,000, and the sample size is calculated to be 727.

Before recruiting college students, the consent of the relevant school leaders and organizations was obtained, and the survey was conducted in a fixed classroom. Based on the consent of college students, we collected data through face-to-face interviews using a questionnaire (Appendix 1). Include students from this school who have communication skills and are willing to participate. Exclude those with poor compliance and those who quit halfway. A total of 821 college students were recruited for this study from August 2023 to April 2024, and the total number of valid participants was 807. After sorting and summarizing the collected questionnaires and eliminating those with missing values and invalid questionnaires (including grade errors, single scale options, or unanswerable questions, etc.), a total of 774 valid questionnaires were obtained, with a questionnaire response rate of 95.91%.

### 2.2 Participants

All participants participated in our study voluntarily, and before the questionnaire, they were informed of the purpose, significance, process, and use of this study. Meanwhile, all survey data were collected anonymously, which was completed in their school. If participants indicate that they cannot continue to participate in the process of filling in the questionnaire, they can withdraw at any time.

### 2.3 Measurements

#### 2.3.1 Social support

Blumenthal created the Perceptive Social Support Scale (PSSS) in 1987 (Blumenthal et al., 1987). The Zimetm Perceptive Social Support Scale (PSSS) (Zimet et al., 1988) was translated and modified into a Chinese version (Chou, 2000; Chen et al., 2018), and this was back translated by a bilingual professional translator Perceptive Social Support Scale consists of 12 items and 3 subscales, including family support, friend support, and significant others. It is used to measure the degree of an individual’s feeling of support from family, friends, and significant others. Each item adopts a seven-point Likert scale ranging from 1 = strongly disagree to 7 = strongly agree. Scores between 12 and 36 indicate a low support level; The intermediate support state is defined as a total score between 37 and 60; The overall score between 61 and 84 denotes strong support. The higher the total score, the higher the individual’s social support. The Cronbach *α* coefficient for the scale was 0.88.

#### 2.3.2 e-Health literacy

The e-Health literacy scale (e-HEALS), developed by Norman and Skinner (2006b), was the first self-reported tool to measure e-Health literacy. At present, e-HEALS is the most widely used electronic health literacy measurement tool. In 2013, the researcher translated the e-HEALS edited by Norman and Skinner into Chinese from the English version (Guo et al., 2013), and formed three dimensions: application ability of network health information and service (1–5 items), evaluation ability (6–7 items), and decision-making ability (8 items), with a total of 8 items. A Likert 5-level scoring method was adopted for each item: “strongly disagree,” “disagree,” “no idea,” “agree,” and “strongly agree” were calculated as 1–5 points, respectively, with a total score of 8–40 points. The higher the score, the better the e-Health literacy. The Cronbach *α* coefficient for the scale was 0.85.

#### 2.3.3 Sleep quality

The Pittsburgh Sleep Quality Index (PSQI) was compiled in Buysse et al. (1989), a psychiatrist from the University of Pittsburgh, and was used to assess the sleep quality of the subjects in the past month. The Chinese version of PSQI translated by Zheng was adopted in this study (Liu et al., 1996). The Chinese versions of the PSQI have been well-validated. It consists of 24 items, of which 19 are self-reported items and 5 other items require ratings from a bed partner. Since the 19th self-reported item and 5 other reported items in the scale do not participate in scoring, only 18 self-reported items that participate in scoring are tested. The results of these 18 test items can be integrated into the 7 components of sleep quality. According to the calculation of rules, each component is scored from 0 to 3 points. The cumulative score of each component is the total score of PSQI. The total score of PSQI ranges from 0 to 2 L, with higher scores indicating poorer sleep quality. The Cronbach *α* coefficient was 0.89.

#### 2.3.4 Internet addiction disorder

Based on the Internet Addiction Screening Scale (a total of 8 items) compiled by gambling addiction identification criteria in the *Diagnostic and Statistical Manual of Mental Disorders-IV* (*DSM-IV-TR*) (American Psychiatric Association, 2013), Young added 12 items and compiled a total of 20 items of Internet Addiction Scale (IAT) (Young, 1998) that is a 5-point Likert scale from 1 (rarely) to 5 (always) and the range of total score is 20–100, which score below 50 points is normal Internet users, the higher the total score, the more serious the degree of Internet addiction disorder, In the Chinese version of IAT (Chang and Man Law, 2008), Items were translated with a focus on teenage relevance. In Item 2, “household chores” was translated as “daily hassles,” while “intimacy with partner” was translated as “activities with companions” in Item 3. Young categorizes respondents who scored under 80 as having a severe Internet addiction disorder and major life issues as a result of their excessive Internet use. The Cronbach’s alpha value was 0.82 in our study.

### 2.4 Statistical analyses

This study employed a structural equation model (SEM) to examine the mechanism by which social support affects Internet addiction disorder, with a focus on the parallel mediating role of electronic health literacy and sleep quality. Based on theory, a multiple mediation model is constructed: two parallel paths are set (social support → e-Health literacy → Internet addiction disorder; Social support → sleep quality → Internet addiction disorder), while age, gender, and other variables were included as covariates in the control. The effects of all paths in the mediation model were evaluated by bootstrap, in which 1,000 repeated samples were conducted, and the 95% Bias-Corrected confidence interval (Bias-corrected CI) is calculated. Statistics were calculated using STATA 16.0. In descriptive analysis categorical variables were described using numbers and percentages, while continuous data were presented as mean ± standard deviation (S.D.). Independent samples t-tests or analysis of variance were used for univariate analysis of variables. And Pearson Product–Moment Correlation Coefficient is used as correlation analysis. Amos 24.0 was used to construct multiple mediation models and path tests. Harman’s single-factor test was used SPSS.

## 3 Results

### 3.1 Participant characteristics

This study first analyzed the influence of common method variance (CMV) through Harman’s single-factor test. The results showed that in the variance explained rate of the unrotated factor, the variance of the first factor accounted for 25.356%, which was below the upper limit of 40%. Table 1 reports sociodemographic characteristics, with more females (62.53%) than males (37.47%). There were 433 (55.94%) students in the self-assessment results who thought their performance was in the average range, and the number of students who thought their performance was good was similar to the number of students who thought their performance was poor. Un-rural and rural students accounted for 38.24 and 61.76%, respectively. The distribution of fathers’ education level is similar in different degrees, while the mother’s education level is mainly concentrated at the junior high school level. In the self-rated family economic status, 64.47% of the students think their family economic status is average. In the self-perceived health report, the proportion of those who think their health is good, average, and bad is 58.53, 36.69, and 4.78%, respectively. The mean social support score was 58.71 (SD = 0.54), the mean e-Health literacy score was 29.17 (SD = 0.24), the sleep quality score was 5.27 (SD = 0.11), and the baseline score for Internet addiction disorder was 38.16 (SD = 0.48). Further, the different groups of gender and self-rated health score differences in Internet addiction disorder were statistically significant.

**Table 1:** Descriptive statistics of sociodemographic characteristics of the study participants.

Variables	*N* (%)	Internet addiction disorder	
		Mean ± SE	*t/F*
Gender			
Male	290 (37.47)	38.31 ± 0.89	0.23
Female	484 (62.53)	38.08 ± 0.57	
Academic record			
Good	155 (20.03)	39.79 ± 1.13	1.32
Fair	433 (55.94)	37.94 ± 0.62	
Poor	186 (24.03)	37.61 ± 1.06	
Birthplace			
Un-rural	296 (38.24)	38.41 ± 0.85	0.40
Rural	478 (61.76)	38.01 ± 0.59	
Fathers’ education			
Primary school and below	183 (23.64)	38.69 ± 0.96	0.32
Junior high school	308 (39.80)	38.28 ± 0.77	
High school and above	283 (36.56)	37.70 ± 0.82	
Mothers’ education			
Primary school and below	256 (33.07)	39.53 ± 0.85	2.23
Junior high school	440 (56.85)	37.66 ± 0.61	
High school and above	78 (10.08)	36.49 ± 1.79	
Family economic status			
Good	80 (10.34)	35.76 ± 1.54	2.65
Fair	499 (64.47)	37.94 ± 0.57	
Poor	195 (25.19)	39.72 ± 1.09	
Self-rated health			
Good	453 (58.53)	36.53 ± 0.59	11.27*
Fair	284 (36.69)	39.81 ± 0.80	
Poor	37 (4.78)	45.54 ± 3.38	

*p < 0.001.

### 3.2 Associations between social support, e-Health literacy, sleep quality and Internet addiction disorder in college students

The results of Pearson correlation coefficients of social support, e-Health literacy, sleep quality, and Internet addiction disorder are presented in Table 2. Results show that social support, e-Health literacy, poor sleep quality, and Internet addiction disorder are negatively related to each other.

**Table 2:** Correlation between social support, e-Health literacy, sleep quality and Internet addiction disorder.

Variables	Social support	e-Health literacy	Sleep quality	Internet addiction disorder
Social support	1.000			
e-Health literacy	0.258*	1.000		
Sleep quality	0.302*	0.196*	1.000	
Internet addiction disorder	−0.192*	−0.152*	0.322*	1.000

*p < 0.001.

### 3.3 Structural model and bootstrap test

**Figure 3:**
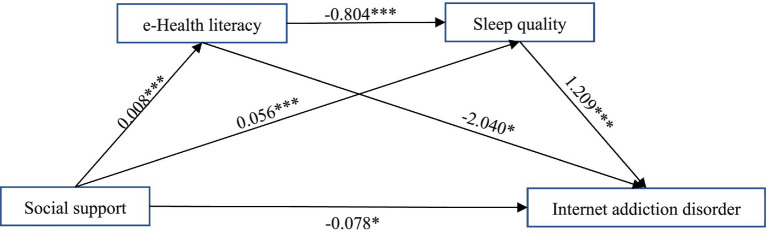
The multiple mediation of e-Health literacy and sleep quality between social support and Internet addiction disorder. *p < 0.05, **p < 0.01 ***p < 0.001.

Internet addiction disorder was the dependent variable, while social support, e-Health literacy, and sleep quality were used as independent variables. All paths in the research model were statistically significant, as shown in Figure 3, which supports all the assumptions. And the results showed that CMIN/DF = 3.985 5; GFI = 0.952; TLI = 0.760; AGFI = 0.938; CFI = 0.964; and SRMR = 0.010, 0.08 were all greater than 0.9. As a result, the data and model were very closely matched.

In Table 3, Social support was negatively associated with Internet addiction disorder, with a significant total effect (estimate = −0.172, *p* < 0.001), and the direct effect accounts for 45.6% of the total effect. Social support was positively associated with e-Health literacy (estimate = −0.008, *p* < 0.001). Social support was associated with lower sleep quality (estimate = −0.056, *p* < 0.001), indicating better actual sleep quality. Higher e-Health literacy was also associated with lower sleep quality scores (estimate = −0.804, *p* < 0.001). The indirect effect of social support on sleep quality through e-Health literacy was significant (estimate = −0.007, *p* = 0.002), confirming the positive influence of e-Health literacy on sleep. The total indirect effect of social support on Internet addiction was significant (estimate = −0.093, *p* < 0.001, 95% CI = [−0.125, −0.062]), accounting for 54.40% of the total effect. All three specific indirect pathways were significant: (1) via sleep quality alone (estimate = −0.068, *p* < 0.001, 95% CI [−0.094, −0.042], 39.45%); (2) via e-Health literacy alone (estimate = −0.017, *p* = 0.038, 95% CI [−0.034, −0.001], 10.12%); and (3) via the serial pathway of e-Health literacy and sleep quality (estimate = −0.008, *p* = 0.007, CI [−0.014, −0.002], 4.83%). Notably, poorer sleep quality (higher scores) was positively associated with Internet addiction (estimate = 1.209, *p* < 0.001), consistent with theoretical expectations.

**Table 3:** Standardization effect and direct effect in the model.

Variables	Standardized estimate	*p*	95%*CI*	Ratio of effect
Social support → e-Health literacy	0.008	<0.001	(0.006, 0.011)	
Social support → Sleep quality	−0.056	<0.001	(−0.074, −0.039)	
e-Health literacy → Sleep quality	−0.804	<0.001	(−1.256, −0.354)	
e-Health literacy → Internet addiction disorder	−2.040	0.035	(−3.932, −0.149)	
Sleep quality → Internet addiction disorder	1.209	<0.001	(0.821, 1.598)	
e-Health literacy → Sleep quality → Internet addiction disorder	−0.973	0.005	(−1.646, −0.301)	
Social support → e-Health literacy → Sleep quality	−0.007	0.002	(−0.011, −0.002)	
Direct effect				**45.60%**
Social support → Internet addiction disorder	−0.078	0.041	(−0.153, −0.003)	
Total indirect effect	−0.093	<0.001	(−0.125, −0.062)	**54.40%**
Social support → Sleep quality → Internet addiction disorder	−0.068	<0.001	(−0.094, −0.042)	39.45%
Social support → e-Health literacy → Internet addiction disorder	−0.017	0.038	(−0.034, −0.001)	10.12%
Social support → e-Health literacy → Sleep quality → Internet addiction disorder	−0.008	0.007	(−0.014, −0.002)	4.83%
Total effect	−0.172	<0.001	(−0.248, −0.096)	/

*p < 0.001. Bold values was used to emphasize the result.

## 4 Discussion

In our study, the prevalence of Internet addiction disorder among college students was measured to be 18.60%, which was consistent with previous research (Li et al., 2018; Pan et al., 2020; Xu et al., 2020). Further, we found the prevalence of Internet addiction disorder in males was higher than in females. This was supported by other studies, all uniformly confirming higher rates in males than females (Cernja et al., 2019; Lee, 2006; Nalwa and Anand, 2003; Rigelsky et al., 2021). The findings of the current study suggested that various Internet usage patterns are probably related to the higher risk of Internet addiction disorder (Cernja et al., 2019), so this gender difference may result from the preference and styles in Internet activities (Yeh et al., 2008), such as male college students are more addicted to online games, while female college students are more addicted to shopping or stars activities, and the activity of playing games exhibits more dangers of developing an addiction (Durkee et al., 2012). Self-rated health is very sensitive as a subjective health evaluation index (Jylhä, 2011; Wuorela et al., 2020). We found that participants with better self-rated health had lower Internet addiction disorder scores in our study. It shows that the more serious the Internet addiction disorder, the deeper the harm to health (Cao et al., 2011). Meanwhile, there was also growing evidence that the negative effects of Internet addiction disorder on both physical and mental health have aroused public concern about health (Li et al., 2018; Ostovar et al., 2021; Wisniewski et al., 2015; Wang et al., 2024c).

This study examined the direct and indirect effects of social support on Internet addiction disorder. First, the social support had a direct negative impact on Internet addiction disorder. The findings confirm earlier research between Internet addiction disorder and social support (Wu et al., 2016; Wang et al., 2011). This direct effect value was −0.078, accounting for 45.60% of the total effect, indicating that social support cognition level was an important factor affecting Internet addiction disorder. Social support is an ongoing social collection in which individuals maintain their social identity and receive emotional support, material assistance, services, information, and new social connections (Mo et al., 2018). Less social support is a risk factor for Internet addiction disorder in college students. A possible explanation is that they have a great need to be recognized, valued, and respected, but they have less social support when social resources are relatively scarce, so it is easy to turn to the Internet for social support (Wu et al., 2016; Yeh et al., 2008), especially if environmental changes force them to restore social networks and communicate with others (Zhang et al., 2018). Instead, they seek social support, satisfaction, and then create a new persona through the Internet (Aspden and Katz, 1998; Di Gennaro and Dutton, 2007; Kang et al., 2013; Zhang et al., 2018). However, some researchers believe that pathological Internet users rarely use the Internet as a tool to search for information, and that relationships formed on such networks are superficial, illusory, and sometimes dangerous and hostile (Fusco et al., 2011; Hills and Argyle, 2003). In addition, some researchers have shown that companionship from supportive people may also reduce their boredom and loneliness, thus reducing their chances of Internet addiction disorder (Mo et al., 2018). Therefore, the higher the level of social support of college students, the more opportunities to provide them with information or emotional support, which their various deeds can be met by offline social support, thus reducing the prevalence of their Internet addiction disorder. This finding has significant implications not only for understanding the causes of Internet addiction disorder but also for understanding the decline of Internet addiction disorder due to social support (Wang and Wang, 2013).

Second, e-Health literacy played a single mediating role between social support and Internet addiction disorder, and the indirect effect value accounted for 10.12% of the total effect value. As a result of the broad access to the Internet and mobile devices, most of the population can access the Internet to search for health-related information. Some studies have shown that individuals with high e-Health literacy are more efficient at finding health information and using health applications, employing more search strategies than those with low e-Health literacy (Neter and Brainin, 2012; Pan et al., 2020; Tennant et al., 2015). There were few articles to investigate the correlation between e-Health literacy and Internet addiction disorder amongst university students. But according to some research findings, e-Health literacy may influence individuals’ health behaviors. For example, individuals with high e-Health literacy may adopt more beneficial health behaviors. Personal exercise habits and eating/food consumption habits can be affected by the utilization of health information on the Internet (Hsu et al., 2014; Mitsutake et al., 2012). Therefore, to help manage their health by improving e-Health literacy has become more important (Norman, 2012; Park, 2019). The presence of social support in college students’ lives also has an impact on e-Health literacy. But studies have shown that even though most college students are competent in computer use and Internet searching, they have varying success rates in finding specific health information because of the credibility and accuracy of the information (Hansen et al., 2003). On one hand, individuals are confronted with an overwhelming volume of health information, yet the sheer abundance of this content can precipitate cognitive overload (Pang and Zhao, 2025). When navigating a vast landscape of contradictory or even misleading health information, even technologically proficient users may experience decision paralysis—paradoxically, the more information available, the harder it becomes to reach a sound conclusion (Ubel, 2002). On the other hand, contemporary college students’ use of technology primarily centers on entertainment, social networking, and basic information retrieval, rather than on the critical evaluation of information, leaving them ill-equipped to discern the authenticity of the information they encounter (Metzger et al., 2003). Furthermore, the digital health information space is heavily saturated with commercial agendas, pseudoscientific marketing, and deliberately misleading content (Huang et al., 2026). For instance, numerous health websites, mobile applications, and social media accounts operate under the guise of “science popularization” while serving underlying product promotion motives. Consequently, while college students may be adept at using digital tools, their capacity to identify and navigate complex commercial. So, social support from family, friends, and classmates, and so on, can provide support in choosing accurate and reliable information (Arcury et al., 2020) to truly assess and evaluate information online (Briones, 2015), as well as improve e-Health literacy.

Third, sleep quality also played an independent mediating role between social support and Internet addiction disorder, accounting for 39.45% of the total effect value. Compared with another indirect path, the indirect effect value is relatively large; this may be related to students’ academic arrangement; they mostly focus on the Internet at night, so changing their sleep habits can directly control and reduce the time spent online, as well as Internet addiction disorder. Previous studies on the sleep issues of college students revealed a strong link between sleep disturbance and Internet addiction disorder (Jahan et al., 2019; Wang et al., 2021; Zhang et al., 2017). Furthermore, a study tested the two-way relationship between Internet addiction disorder and sleep quality, and the results were statistically significant (Jiang et al., 2022). We also found that social support affected sleep quality, which is consistent with previous studies (Chen, 2018; van Schalkwijk et al., 2015). Higher social support means that there are more opportunities for information and intervention for college students. In addition, other research has shown that subjective perceptions of sleep quality are related to an individual’s attitude toward sleep (Peach et al., 2018). Thus, good social support helps to promote the cycle of higher sleep quality-lower Internet addiction disorder, and good health.

Finally, e-Health literacy and sleep quality played serial-multiple mediating roles in social support and Internet addiction disorder, accounting for 4.83% of the total effect, which is the first study to investigate a serial-multiple mediation model of the relationships between social support, e-Health literacy, sleep quality, and Internet addiction disorder in college students. The results in our study indicated that social support affected e-Health literacy, and then e-Health literacy affected Internet addiction disorder through the path of sleep quality. A possible explanation is that social relationships will provide college students with various health-related information and he hazards of a bad behavioral lifestyle, which can promote their search and identification of relevant information through the Internet, while the Internet will calculate and push individual information preferences based on users’ search, called “recommendation algorithm” (Kong et al., 2021; Resnick, 1997). So, the more they have access to health-related information when they use the Internet. According to the KAP theory (Cust and Finch, 1963) and Health Belief Model (HBM) (Janz and Becker, 1984), the stimulation of health information prompts the production of a change of attitudes and beliefs of the foundation, and then the individual perceives the threat of the disease and believes that they can prevent the disease by taking certain actions. In general, the more social support acquired by individuals acquire, the stronger their tendency to change their bad behavioral lifestyle. In our research, we have expanded the possible research perspective for the related research of Internet addiction disorder in the context of Internet modernization, and through the study of Chinese college students as an Internet user group, it is helpful to further understand the complex relationship between social support, e-Health literacy, sleep quality and Internet addiction disorder, and help to determine the intervention plan for the prevention and treatment of Internet addiction disorder.

### 4.1 Limitations

There were also some limitations to our study. First, our study was a cross-sectional survey, and the causal relationship between variables could not be determined. Second, participants were all from Nantong, which may affect the generalizability of the results. For example, social and cultural differences across various regions, as well as the rules and regulations of different schools, etc. Third, other unmeasured potential factors may have confounded the results, such as family situation, childhood experiences, and living environment, etc. Future research can address these limitations to advance the understanding of Internet addiction disorder among university students. Tracking students longitudinally could reveal how changes in social support led to improvements in e-Health literacy and sleep quality, ultimately reducing Internet addiction disorder symptoms. Expanding the sampling framework to include more diverse geographical locations (e.g., universities from eastern, central, and western China) and different types of institutions (e.g., vocational colleges) would enhance the external validity of the findings. Furthermore, future studies could incorporate more objective measures to complement self-reports. For instance, actigraphy or polysomnography could provide objective data on sleep patterns.

### 4.2 Policy implications

Our findings suggest that effective strategies should move beyond merely restricting internet use. Instead, several practical ways to reduce Internet addiction among university students. First, universities can strengthen social support systems by implementing mentorship programs, peer support groups, and community-building activities that enhance students’ sense of belonging and connection—both online and offline. Second, institutions should integrate e-Health literacy education on teaching e-health skills into curricula, teaching students how to efficiently find, evaluate, and apply online health information. This empowers them to use the internet as a tool for wellbeing rather than a source of addiction. Third, sleep health programs can be promoted via sleep hygiene education, environmental changes (e.g., quiet hours, tech-free zones), and credit-bearing courses on sleep science. Finally, these efforts should be combined within a multi-component framework that builds students’ capability, opportunity, and motivation for healthy technology use. Universities and policymakers could consider e-Health literacy education, funding sleep initiatives, and regulating misleading online health information to help students navigate the digital world without harming their wellbeing.

## 5 Conclusion

This study established and validated a multiple mediation model, demonstrating that social support reduces Internet Addiction Disorder (IAD) among Chinese university students by enhancing their e-Health literacy and improving sleep quality. These findings reveal that robust social support not only provides emotional comfort but also equips students with the skills to navigate digital health information and fosters healthier sleep patterns, collectively mitigating Internet Addiction Disorder risk. From a practical standpoint, this research offers a multifaceted framework for developing targeted interventions aimed at preventing and reducing internet addiction disorder in the university population. Our findings suggest that effective strategies should move beyond merely restricting internet use. Instead, we should adopt a holistic approach, such as strengthens the social support system; integrates e-Health literacy education and promotes sleep hygiene and so on.

## Data Availability

Data are available from the corresponding author on reasonable request.
