# Supplementary material for: The relationship between social support, e-Health literacy, sleep quality and Internet addiction disorder in college students: a cross-sectional study
Source: Front Psychol. 2026 Apr 7;17:1754039. doi: 10.3389/fpsyg.2026.1754039 (PMC13095773; doi:10.3389/fpsyg.2026.1754039)
Supplement: Supplementary file 1 [file Table_1.DOCX]

**Survey on the** **Health Status of College students**

We are members of the research group "Health status of college students" of Dongying People’s Hospital (Dongying Hospital of Shandong Provincial Hospital Group). To understand the health status of college students, we conducted this survey. This survey is anonymous, and your information is only used for research analysis. Thank you for your cooperation!

**A. Basic information**

|  | **Questions and choices** |  |
| --- | --- | --- |
| **A01** | **Gender:** ①Male ②Female |  |
| **A02** | **What is your grade?**  ①First-year ②Second-year ③Third-year ④Fourth-year ⑤Fifth-year |  |
| **A03** | **Major:** |  |
| **A04** | **What is your academic in your class compared with the classmates?**  ①Very good ②Good ③Average ④Not good ⑤Very bad |  |
| **A05** | **Birth Place:** ①Unrural ②Rural |  |
| **A06** | **What is your father's education?**  ①Illiterate ②Elementary school ③Middle school ④High school/vocational school  ⑤Two-/Three-Year College/Associate degree ⑥Bachelor's degree and above |  |
| **A07** | **What is your mother's education?**  ①Illiterate ②Elementary school ③Middle school ④High school/vocational school  ⑤Two-/Three-Year College/Associate degree ⑥Bachelor's degree and above |  |
| **A08** | **How do you feel about your family’s financial situation?**  ①Very good ②Good ③Average ④Not good ⑤Very bad |  |
| **A09** | **How do you feel about your health?**  ①Very good ②Good ③Average ④Not good ⑤Very bad |  |

**B. e-Health Literacy**

| **B01** | **I know how to find helpful health resources on the Internet.**  ①Strongly disagree ②Disagree ③Undecided ④Agree ⑤Strongly agree |  |
| --- | --- | --- |
| **B02** | **I know how to use the Internet to answer my health questions.**  ①Strongly disagree ②Disagree ③Undecided ④Agree ⑤Strongly agree |  |
| **B03** | **I know what health resources are available on the Internet.**  ①Strongly disagree ②Disagree ③Undecided ④Agree ⑤Strongly agree |  |
| **B04** | **I know where to find helpful health resources on the Internet.**  ①Strongly disagree ②Disagree ③Undecided ④Agree ⑤Strongly agree |  |

| **B05** | **I know how to use the health information I find on the Internet to help me.**  ①Strongly disagree ②Disagree ③Undecided ④Agree ⑤Strongly agree |  |
| --- | --- | --- |
| **B06** | **I have the skills I need to evaluate the health resources I find on the Internet.**  ①Strongly disagree ②Disagree ③Undecided ④Agree ⑤Strongly agree |  |
| **B07** | **I can tell high quality from low quality health resources on the Internet.**  ①Strongly disagree ②Disagree ③Undecided ④Agree ⑤Strongly agree |  |
| **B08** | **I feel confident in using information from the Internet to make health decisions.**  ①Strongly disagree ②Disagree ③Undecided ④Agree ⑤Strongly agree |  |

**C. Perceived Social Support Scale**

|  |  | Very Strongly Disagree | Strongly Disagree | Mildly Disagree | Neutral | Mildly Agree | Strongly Agree | Very Strongly Agree |  |
| --- | --- | --- | --- | --- | --- | --- | --- | --- | --- |
| **C01** | There is a special person who is around when I am in need. |  |  |  |  |  |  |  |  |
| **C02** | There is a special person with whom I can share my joys and sorrows. |  |  |  |  |  |  |  |  |
| **C03** | My family really tries to help me. |  |  |  |  |  |  |  |  |
| **C04** | I get the emotional help and support I need from my family. |  |  |  |  |  |  |  |  |
| **C05** | I have a special person who is a real source of comfort to me. |  |  |  |  |  |  |  |  |
| **C06** | My friends really try to help me. |  |  |  |  |  |  |  |  |
| **C07** | I can count on my friends when things go wrong. |  |  |  |  |  |  |  |  |
| **C08** | I can talk about my problems with my family. |  |  |  |  |  |  |  |  |
| **C09** | I have friends with whom I can share my joys and sorrows. |  |  |  |  |  |  |  |  |
| **C10** | There is a special person in my life who cares about my feelings. |  |  |  |  |  |  |  |  |
| **C11** | My family is willing to help me make decisions. |  |  |  |  |  |  |  |  |
| **C12** | I can talk about my problems with my friends. |  |  |  |  |  |  |  |  |

**D. Pittsburgh Sleep Quality Index**

| **Part 1** | | | | | | |
| --- | --- | --- | --- | --- | --- | --- |
| **D01** | When have you usually gone to bed? | | | | |  |
| **D02** | How long (in minutes) has it taken you to fall asleep each night? | | | | |  |
| **D03** | When have you usually gotten up in the morning? | | | | |  |
| **D04** | How many hours of actual sleep do you get at night? (This may be different than the number of hours you spend in bed) | | | | |  |
| **Part 2** | | | | | | |
|  | During the past month, how often have you had trouble sleeping because you? | Not during the past month | Less than once a week | Once or twice a week | Three or more times a week |  |
| **D05.1** | Cannot get to sleep within 30 minutes. |  |  |  |  |  |
| **D05.2** | Wake up in the middle of the night or early morning |  |  |  |  |  |
| **D05.3** | Have to get up to use the bathroom. |  |  |  |  |  |
| **D05.4** | Cannot breathe comfortably. |  |  |  |  |  |
| **D05.5** | Cough or snore loudly. |  |  |  |  |  |
| **D05.6** | Feel too cold. |  |  |  |  |  |
| **D05.7** | Feel too hot. |  |  |  |  |  |
| **D05.8** | Have bad dreams. |  |  |  |  |  |
| **D05.9** | Have pain. |  |  |  |  |  |
| **D05.10** | Other reason(s), please describe, including how often you have had trouble sleeping because of this reason(s): |  |  |  |  |  |
| **D06** | During the past month, how often have you taken medicine (prescribed or “over the counter”) to help you sleep? |  |  |  |  |  |
| **D07** | During the past month, how often have you had trouble staying awake while driving, eating meals, or engaging in social activity? |  |  |  |  |  |
| **D08** | During the past month, how much of a problem has it been for you to keep up enough enthusiasm to get things done? |  |  |  |  |  |
| **D09** | **During the past month, how would you rate your sleep quality overall?**  ①Very good ②Fairly good ③Fairly bad ④Very bad | | | | |  |

**E. Internet Addiction Scale**

|  |  | Rarely | Occasionally | Frequently | Often | Always |  |
| --- | --- | --- | --- | --- | --- | --- | --- |
| **E01** | Do you find that you stay online longer than you intended? |  |  |  |  |  |  |
| **D02** | Do you neglect household chores to spend more time online? |  |  |  |  |  |  |
| **D03** | Do you prefer the excitement of the internet to intimacy with your partner? |  |  |  |  |  |  |
| **D04** | Do you form new relationships with fellow online users? |  |  |  |  |  |  |
| **D05** | Do others in your life complain to you about the amount of time you spend online? |  |  |  |  |  |  |
| **D06** | Does your work suffer because of the amount of time you spend online? (E.g.‚ postponing things‚ not meeting deadlines‚ etc.) |  |  |  |  |  |  |
| **D07** | Do you check your email before something else you need to do? |  |  |  |  |  |  |
| **D08** | Does your job performance or productivity suffer because of the internet? |  |  |  |  |  |  |
| **D09** | Do you become defensive or secretive when anyone asks you what you do online? |  |  |  |  |  |  |
| **D10** | Do you block disturbing thoughts about your life with soothing thoughts of the internet? |  |  |  |  |  |  |
| **D11** | Do you find yourself anticipating when you will go online again? |  |  |  |  |  |  |
| **D12** | Do you fear that life without the internet would be boring‚ empty or joyless? |  |  |  |  |  |  |
| **D13** | Do you snap‚ yell‚ or act annoyed if someone bothers you while you are online? |  |  |  |  |  |  |
| **D14** | Do you lose sleep due to late night internet use? |  |  |  |  |  |  |
| **D15** | Do you feel preoccupied with the internet when not online‚ or fantasize about being online? |  |  |  |  |  |  |
| **D16** | Do you find yourself saying “Just a few more minutes” when online? |  |  |  |  |  |  |
| **D17** | Do you try to cut down on the amount of time you spend online and fail? |  |  |  |  |  |  |
| **D18** | Do you try and hide how long you’ve been online? |  |  |  |  |  |  |
| **D19** | Do you choose to spend more time online over spending time out with others? |  |  |  |  |  |  |
| **D20** | Do you feel depressed‚ moody‚ or nervous when you are not online‚ and do these feelings go awhile when you go back online? |  |  |  |  |  |  |
